# Efficacy of subtenon 20-mg triamcinolone injection versus 0.1% dexamethasone eye drops for controlling inflammation after phacoemulsification: a randomized controlled trial

**DOI:** 10.1038/s41598-022-20522-y

**Published:** 2022-10-01

**Authors:** Pitipol Choopong, Nuttacha Taetrongchit, Sutasinee Boonsopon, Atchariya Nimkarn, Kanyapak Srisukkosalin, Pratuangsri Chonpimai, Waree Nujoi, Krissana Maneephagaphun, Noppakhun Panyayingyong, Nattaporn Tesavibul

**Affiliations:** 1 Department of Ophthalmology, Faculty of Medicine, Siriraj Hospital, Mahidol University, 2 Wanglang Road, Bangkok-noi, Bangkok, 10700 Thailand; 2 Department of Ophthalmology, Mettapracharak Hospital, Nakhon Pathom, Thailand

**Keywords:** Randomized controlled trials, Lens diseases

## Abstract

A prospective randomized control trial of 140 eyes from 140 patients, who underwent phacoemulsification, was conducted to compare the efficacy of subtenon corticosteroids injection with corticosteroids eye drops for controlling postoperative intraocular inflammation. Seventy patients received subtenon 20-mg triamcinolone injection (TA group), whereas the other 70 patients received 0.1% dexamethasone eye drops (Dexa group) after the uneventful surgeries. We examined and measured anterior chamber inflammation (ACI) score, laser flare-cell metering, conjunctival redness, pain, discomfort, visual acuity, intraocular pressure, and central foveal thickness on 1, 7, 14, 28 and 90 days postoperatively. At one month after the surgery, full recovery (zero ACI score) was found in 43 patients (63.20%) in the Dexa group versus 47 patients (68.10%) in the TA group (*p* = 0.55). There were no statistically significant differences in aqueous cells (*p* = 0.37) and flare (*p* = 0.86) between the two groups at one month. All participants experienced no serious adverse events. In conclusion, we found no statistically significant difference between subtenon 20-mg triamcinolone injection and 0.1% dexamethasone eye drop to control inflammation postoperatively. A single subtenon 20-mg triamcinolone injection could be an alternative anti-inflammatory treatment for an uneventful phacoemulsification.

## Introduction

Cataract, an opacification of the natural crystalline lens, is a significant cause of reversible blindness worldwide. The only treatment is a surgery to replace the opaque lens with a clear artificial intraocular lens (IOL). The most common procedure for modern cataract surgery is phacoemulsification^1^. Despite the advance in technologies, intraocular inflammation in response to tissue injury always occurs postoperatively. Although the intensity of the reaction is usually mild to moderate, it tends to persist for months without proper anti-inflammatory medications. Prolonged postoperative inflammation leads to ocular discomfort, cystoid macular edema, and glaucoma secondary to inflammation. In addition, persistent intraocular inflammation also hastens posterior lens capsule opacity, affecting patients' visual acuity^2^.

To control postoperative inflammation, surgeons employed many anti-inflammatory agents, including corticosteroids and nonsteroidal anti-inflammatory drugs (NSAIDs), via various routes after the surgery. Topical corticosteroid eye drops, such as 0.1% dexamethasone and 1% prednisolone acetate, are commonly applied for two to four weeks until the intraocular inflammation subsided clinically. While topical NSAIDs had better penetration than corticosteroids, they demonstrated stinging sensation and inferior anti-inflammatory effect^3^. However, the disadvantages of topical eyedrop are short contact time and relatively poor ocular penetration. Moreover, the elderly, who are the majority of patients, may find difficulties in eye drops usage. As a result, this problem could lead to poor compliance and unfavorable outcomes.

A single dose of intraocular injection, including intracameral^4,5^ and intravitreal^6,7^ of triamcinolone acetonide, appears to be promising treatment options for controlling postoperative inflammation following cataract surgery because of its rapid onset and high drug concentration. However, serious complications such as toxic anterior-segment syndrome, corneal endothelial damage, snow-globe effect, glaucoma, and endophthalmitis were reported^8–11^.

In comparison, subtenon injection of depot corticosteroids is easy and safe. It demonstrates balances among anti-inflammation efficacy, complication, and compliance. The procedure allows the medicine to be continuously released into intraocular space. The effect lasts for 4–6 weeks after a single injection, making this procedure possible to replace topical corticosteroids. Therefore, a single subtenon triamcinolone injection could be an alternative treatment to control inflammation after cataract surgery, especially in patients with poor drug compliance^12^. Nevertheless, the slight drawbacks of the procedure are the risks of developing glaucoma and globe penetration.

The previous studies found no differences in postoperative inflammatory control after phacoemulsification between single 25- to 40-mg subtenon triamcinolone injections and various topical corticosteroids^13–16^. However, most studies evaluated the efficacy of treatments by clinical observation of anterior chamber reaction using a slit-lamp biomicroscope. The method qualitatively and subjectively evaluates the inflammation, leading to measurement bias. Instead, the quantitative and objective measurement of postoperative inflammation (flare and cells) using the laser flare-cell meter would be more accurate and reproducible^17^. Furthermore, most studies had short follow-up periods to evaluate the side effects of subtenon corticosteroids.

To date, the lowest dose reported to control postoperative inflammation was a 25-mg subtenon triamcinolone injection. However, with the well-developed surgical techniques and intraocular lens materials, the inflammation would be lesser than in the past. Therefore, the objective of this study was to quantitatively compare the efficacy and safety of a single subtenon injection of 20-mg triamcinolone with 0.1% dexamethasone eye drops for intraocular inflammation control after uneventful phacoemulsification with IOL implantation.

## Methods

### Patient selection

We enrolled the patients scheduled to undergo phacoemulsification with posterior chamber IOL implantation at the Department of Ophthalmology, Siriraj Hospital, from May 2012 to February 2016 to participate in this randomized controlled study. All participants agreed and signed the informed consent before enrollment. This study was performed according to the principles of the Declaration of Helsinki. The study protocol was reviewed and approved by the Siriraj Institutional Review Board. (SIRB), Siriraj Hospital, Mahidol University, Bangkok, Thailand. The IRB number was 180/2555(EC2). The registered number of the clinical trial was NCT01801774 (www.clinicaltrial.gov). The date of first registration was 01/03/2013.

We excluded the patients with the conditions that might interfere postoperative inflammatory results, which were patients with known ocular diseases, including uveitis, and diabetic retinopathy, patients who had undergone previous intraocular procedures, patients with known autoimmune diseases or immune-deficiencies patients and patients who had taken corticosteroids, nonsteroidal anti-inflammatory drugs (NSAIDs), or immunomodulating agents within three months prior to the surgery. Patients who were diagnosed with glaucoma and ocular hypertension would not be able to enroll into the study. We also excluded patients who were allergic to corticosteroids and macrolides, pregnant and lactating women. For patients who developed complications during cataract surgery, such as ruptured posterior capsule with or without vitreous loss and dropped lens nucleus, would be excluded from the study.

### Surgical technique

One surgeon (PC) performed the phacoemulsification with the same surgical technique. Briefly, the patient underwent surgery after the pupil was dilated with 2.5% phenylephrine combined with 0.75% tropicamide eye drop. The eye was anesthetized with 2% xylocaine subconjunctival injection and irrigated with 5% povidone-iodine before the surgery. Phacoemulsification was operated through a temporal 2.4-mm clear corneal incision using a Stellaris® phacoemulsification system (Bausch & Lomb, Rochester, New York, USA). The stop-and-chop technique was the preferred surgical fashion; however other surgical techniques were used depending on the intraoperative findings. The implant was foldable hydrophobic acrylic IOL. After an uneventful surgery, the patient was randomly injected, whether 20-mg triamcinolone or none, through the subtenon route at the inferior fornix using 27G, ½-inch needle and 3-ml syringe to control postoperative inflammation. Then, 20-mg gentamicin was injected subconjunctivally in both groups to prevent postoperative infection. One drop of levofloxacin eye drop was used as an immediate postoperative antibiotic.

### Treatment assignment and masking

This randomized controlled trial was the parallel group, two-armed trial with 1:1 allocation ratio. The treatment assignment was performed using the block-of-six randomization based on computer-generated random numbers. The assignment letters were sequentially concealed in the sealed and opaque envelopes by two investigators (AN and SB). After an uneventful surgery, an investigator (KS) disclosed the treatment assignment to the surgeon. The patients were randomly received whether subtenon triamcinolone injection (TA group) or topical dexamethasone eye drop (Dexa group). In the TA group, the patients received a 20-mg triamcinolone acetonide subtenon injection intraoperatively. To mask the patients and to prevent postoperative infection, both groups received 20-mg gentamicin subconjunctival injection at the end of the surgery.

Postoperatively, the patients in TA group received 0.3% tobramycin eye drop (Tobrex®, Alcon, Fort Worth, Texas, USA) 4 times a day for 28 days. In the Dexa group, the patient received a combined 0.1% dexamethasone/0.3% tobramycin eye drop (Tobradex®, Alcon, Fort Worth, Texas, USA) 4 times a day for 28 days starting at the first postoperative day. The investigators (PC, WN, KM) performed the clinical evaluations and flare-cell metering without knowing the patient's information and the treatment assigned.

### Outcome measures

All patients were evaluated prior to surgery and 1, 7, 14, 28, and 90 days postoperatively (PODs 1, 7, 14, 28, and 90). At each visit, the investigators recorded ocular symptoms and signs (pain, discomfort, and conjunctival injection), best-corrected visual acuity (BCVA), intraocular pressure (IOP) in mmHg using Goldmann applanation tonometry, slit-lamp biomicroscopy, central foveal thickness (CFT) using spectral-domain optical coherence tomography (OCT), and a laser flare-cell metering (LFCM) with the FC-1000 laser flare-cell meter (Kowa, Japan) of the participants. Dilated fundoscopic examination was performed at baseline, 28, and 90 days postoperatively to evaluate cystoid macular edema and other adverse events such as retinal break and retinal detachment.

The patients graded their symptoms in terms of pain and discomfort with a 5-scale system (0 = none, 1 = mild, 2 = moderate, 3 = severe, and 4 = very severe). Eye discomfort was the feeling of ocular unease such as foreign body sensation, irritation, and photophobia. The investigators also evaluated conjunctival injection in a similar grading. The anterior chamber reaction was assessed qualitatively and quantitatively. The qualitative assessment was done under slit-lamp biomicroscopic examination. We graded anterior chamber cells and flare according to the Standardization of Uveitis Nomenclature (SUN) working group criteria using 1 mm × 1 mm slit beam^18^. The anterior chamber cell was graded on a scale of 0 to 4, where grade 0 is no cell, grade 0.5 is 1–5 cells, grade 1 is 6–15 cells, grade 2 is 16–25 cells, grade 3 is 26–50 cells and grade 4 is 50 or more cells. The anterior chamber flare was graded on a scale of 0 to 4, where grade 0 is no Tyndall effect, 1 is faint Tyndall effect, 2 is moderate Tyndall effect, 3 is marked Tyndall effect and 4 is intense Tyndall effect. We used the anterior chamber inflammation (ACI) score, which is a summation of anterior chamber cells and flare grading, for statistical analyses^19^. The quantitative measurement of anterior chamber reaction was performed using LFCM and was reported in cells/mm^3^ for cell metering and photons/millisecond for flare metering.

### Statistical analysis

With a power of 80% and a type one error rate of 0.05, we estimated the minimum of 62 patients per group to detect a 20% difference of proportion of patients with zero ACI score within 28 days between the two groups. With an estimated 10% dropout rate, a final sample size of 70 patients per group was required. An intention-to-treat analysis was performed. Missing data were imputed with the last observation data carried forward. Descriptive statistics were used to describe baseline characteristics and adverse events. The Chi-square test was used to evaluate the differences in the proportion of complete resolution (ACI score = 0) between groups on POD28.

We compared mean differences in visual acuity, intraocular pressure, and anterior chamber laser cell and flare between groups at each time point using independent t-tests. Non-parametric tests were used for non-normally distributed data. We set a significance level of 0.05 to determine differences in outcomes. All statistical analyses were performed using SPSS Statistics v.18 (SPSS, Chicago, Illinois, USA).

## Results

**Figure 1:**
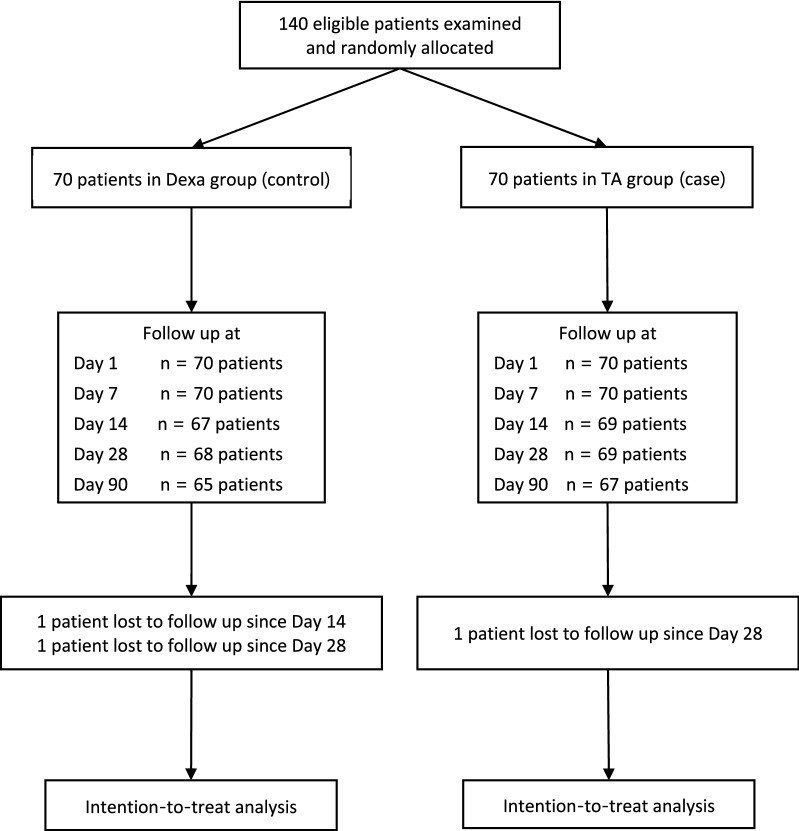
Flow diagram of patients through the study.

A total of 140 eyes from 140 patients underwent phacoemulsification with posterior chamber IOL implantation from May 2012 to February 2016 were enrolled in this study. There were 81 right eyes, 59 males, and the mean age of patients was 68.3 ± 8.8 years old. The participants were randomly assigned into two equal groups (Dexa group or TA group). Seven patients in the Dexa group and five patients in the TA group missed at least one appointment due to a lack of caregivers or personal illness not related to treatments (Fig. 1). However, only two patients in the Dexa group and one patient in the TA group missed the POD28 examination. Baseline characteristics of the participants in each group were listed in Table 1. The TA group included slightly more male patients. There was no disparity between both groups in other characteristics, including age, laterality, BCVA, IOP, LFCM, and CFT at the baseline. There was no difference in operating time between the two groups, although the TA group had twice more patients with dense cataract (12.9% in TA group vs 7.1% in Dexa group).

**Table 1:** Baseline demographic characteristics of the patients in comparison groups.

Baseline	Dexa group	TA group
	(n = 70)	(n = 70)
Mean age in years ± SD	68.41 ± 7.91	68.21 ± 9.61
Male, n (%)	23 (32.9)	36 (51.4)
Right eye, n (%)	39 (55.7)	42 (60.0)
**Nuclear sclerosis grading, n (%)**		
No nuclear sclerosis	2 (2.9)	1 (1.4)
1 +	28 (40.0)	30 (42.9)
2 +	35 (50.0)	30 (42.9)
3 +	4 (5.7)	7 (10.0)
4 +	1 (1.4)	2 (2.9)
Mean BCVA in logMAR* ± SD	0.39 ± 0.25	0.40 ± 0.39
Mean IOP in mmHg* ± SD	14.21 ± 3.41	13.77 ± 2.80
Mean Cells/mm^3^* ± SD	8.63 ± 14.72	9.60 ± 21.85
Mean Flare (photons/ms)* ± SD	4.66 ± 2.34	4.87 ± 3.43
Mean CFT (microns)* ± SD	229.54 ± 25.80	243.81 ± 93.79
Mean Phaco time in minute ± SD	0.60 ± 0.48	0.77 ± 0.79

Dexa group = 0.1% dexamethasone eye drop group; TA group = subtenon 20-mg triamcinolone injection group.

*BCVA* best-corrected visual acuity, *logMAR* logarithm of the minimum angle of resolution, *IOP* intraocular pressure, *CFT* central foveal thickness, *Phaco* phacoemulsification, *SD* standard deviation.

*Preoperative measurement.

### Clinical evaluations

**Figure 2:**
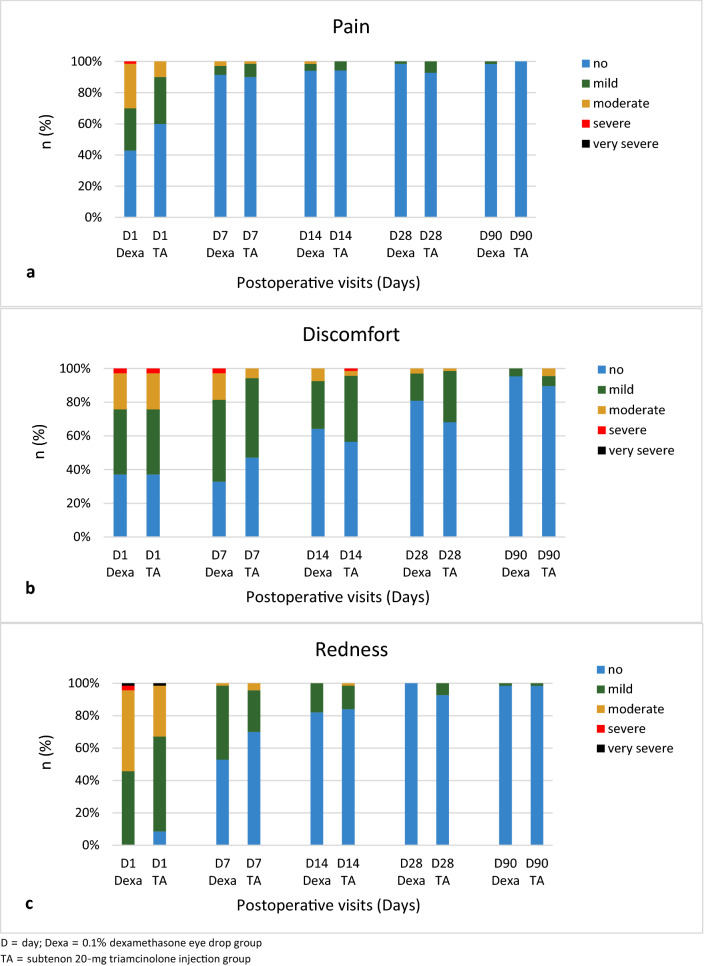
Grades of pain (**a**), discomfort (**b**), and redness (**c**) at each study visit. Mild to moderate pain, discomfort, and redness were observed during early postoperative period and gradually subsided.

Mild to moderate pain and discomfort were reported during the early postoperative period, which was gradually subsided in both groups (Fig. 2a, b). One patient (1.4%) in the Dexa group stated severe pain on POD1, which rapidly decreased in the subsequent visits. Two of each group (2.9%) demonstrated severe ocular discomfort on POD1, which faded eventually. The conjunctival redness demonstrated similar trends (Fig. 2c).

### ACI score and laser flare-cell meter

**Figure 3:**
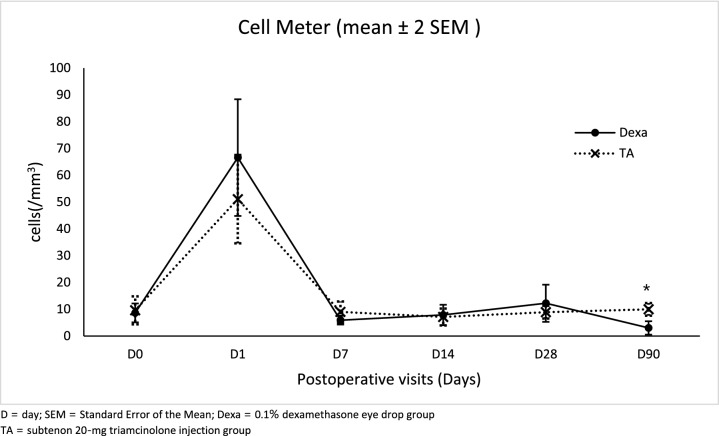
Mean anterior chamber cell ± 2 SEM (/mm^3^) from laser flare-cell meter at each study visit. The mean cells of Dexa group were significantly lower than TA group at post operative day 90 (mean difference −6.93; 95% CI −10.34, −3.52; *p < 0.001).

**Figure 4:**
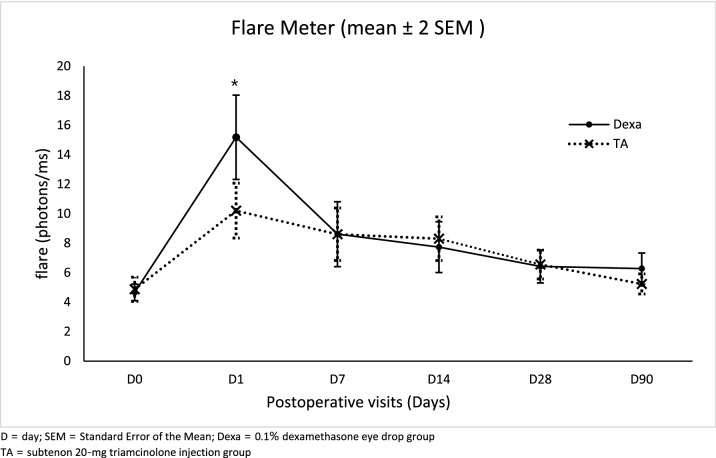
Mean anterior chamber flare ± 2 SEM (photons/ms) from laser flare-cell meter at each study visit. The mean flare of TA group was significantly lower than Dexa group on post operative day 1 (mean difference 4.98; 95% CI 1.59, 8.36; *p = 0.004).

The ACI scores of each visit were shown in Table 2. The proportions of patients who had zero ACI score were comparable between both groups in every visit. No statistically significant differences were observed. Cells and Flare measurement from LFCM demonstrated the most intense inflammation on POD1, and the reaction subsided to baseline eventually. The mean flare was significantly lower in the TA group than the Dexa group on POD1 (mean difference 4.98; 95% CI 1.59, 8.36; *p* = 0.004), whereas the Dexa group had significantly lower cells than the TA group at POD90 (mean difference −6.93; 95% CI −10.34, −3.52; *p* < 0.001). Besides, there were no significant differences of anterior chamber cells and flare over the course between treatment groups (Figs. 3 and 4).

**Table 2:** Proportion of zero anterior chamber inflammation (ACI) score at each study visit.

Postoperative date	Zero ACI score in Dexa group	Zero ACI score in TA group	RR (95%CI)	*p* value
	n (%)	n (%)		
Day 1	0	0	–	–
Day 7	11 (15.7)	7 (10.0)	1.57 (0.65, 3.82)	0.31
Day 14	19 (28.4)	21 (30.4)	0.93 (0.55, 1.57)	0.71
Day 28	43 (63.2)	47 (68.1)	0.93 (0.73, 1.18)	0.55
Day 90	56 (86.2)	59 (88.1)	0.98 (0.86, 1.12)	0.11

No difference in proportion of patients who had zero ACI score was noted in any visit between two groups.

Dexa group = 0.1% dexamethasone eye drop group.

TA group = subtenon 20-mg triamcinolone injection group.

*ACI* = anterior chamber inflammation.

*RR* relative risk, *CI* confidence interval.

### Intraocular pressure, BCVA and other adverse events

**Figure 5:**
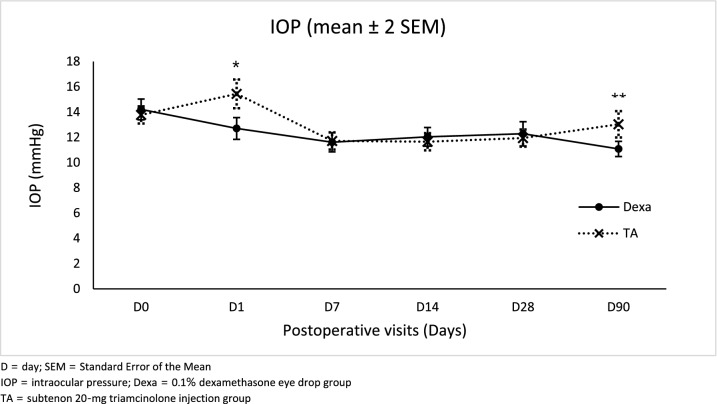
Mean IOP (mmHg) ± 2 SEM at each study visit. Mean IOP in Dexa group was significantly lower than in TA group on POD1 (mean difference −2.74, 95% CI −4.15, −1.34; *p < 0.001) and post operative day 90 (mean difference −1.95, 95% CI, −3.15, −0.76; **p = 0.002). Whereas, there was no significant difference in mean IOP between treatment arms in the other visits.

Patients in the Dexa group demonstrated significantly lower IOP than those in the TA group on POD1 (mean difference −2.74, 95% CI −4.15, −1.34; *p* < 0.001) and POD 90 (mean difference −1.95, 95% CI, −3.15,   −0.76; *p* = 0.002). However, there was no significant difference in mean IOP between treatment arms in the other visits (Table 3, Fig. 5). One patient in the Dexa group and three patients in the TA group experienced transient equal to or higher than 25 mmHg IOP at least once during follow-up visits. Their IOPs were controlled under temporary uses of anti-glaucoma eye drops. BCVA did not show statistically significant difference between groups in each visit. No CFT change was observed in all cases (Table 3). Two patients in the TA group demonstrated pigment dispersion. No one in the study experienced injection-related complications, cystoid macular edema, or endophthalmitis.

**Table 3:** Intraocular pressure (IOP), best-corrected visual acuity (BCVA), and central foveal thickness (CFT) at each study visit.

Variables	Groups	Postoperative date					
		Day 0	Day 1	Day 7	Day 14	Day 28	Day 90
IOP (mmHg)	Dexa group (mean ± SD)	14.21 ± 3.41	12.70 ± 3.60	11.61 ± 3.21	12.04 ± 3.06	12.28 ± 3.92	11.08 ± 2.45
	TA group (mean ± SD)	13.77 ± 2.80	15.44 ± 4.73	11.71 ± 2.85	11.65 ± 2.84	11.94 ± 2.83	13.03 ± 4.28
	Mean difference (95%CI)	0.44 (−0.60, 1.49)	−2.74 (−4.15, −1.34)	−0.10 (−1.11, 0.91)	0.39 (−0.61, 1.39)	0.34 (−0.82, 1.49)	−1.95 (−3.15, −0.76)
	*p* value	0.4	< 0.001	0.85	0.44	0.56	0.002
BCVA (logMAR)	Dexa group (mean ± SD)	0.39 ± 0.25	0.18 ± 0.12	0.16 ± 0.11	0.14 ± 0.09	0.14 ± 0.11	0.13 ± 0.10
	TA group (mean ± SD)	0.40 ± 0.39	0.18 ± 0.16	0.13 ± 0.10	0.13 ± 0.11	0.11 ± 0.09	0.12 ± 0.09
	Mean difference (95%CI)	−0.01 (−0.12, 0.10)	0.00 (−0.05, 0.05)	0.02 (−0.01, 0.06)	0.01 (−0.02, 0.04)	0.03 (−0.01, 0.06)	0.01 (−0.02, 0.04)
	*p* value	0.87	0.92	0.19	0.58	0.1	0.45
CFT (microns)	Dexa group (mean ± SD)	229.54 ± 25.80	226.10 ± 23.35	225.35 ± 25.03	226.73 ± 25.51	233.31 ± 26.42	234.33 ± 25.90
	TA group (mean ± SD)	243.81 ± 93.79	227.24 ± 28.61	225.67 ± 28.27	234.72 ± 53.34	236.81 ± 31.20	236.43 ± 32.35
	Mean difference (95%CI)	−14.27 (−37.26, 8.72)	−1.13 (−10.03, 7.77)	−0.32 (−9.28, 8.64)	−7.99 (−22.24, 6.26)	−3.50 (−13.31, 6.31)	−2.10 (−12.31, 8.11)
	*p* value	0.22	0.8	0.94	0.27	0.48	0.69

IOP = intraocular pressure, BCVA = best-corrected visual acuity, CFT = central foveal thickness.

logMAR = logarithm of the minimum angle of resolution; SD = Standard Deviation; CI = Confidence Interval.

Dexa group = 0.1% dexamethasone eye drop group; TA group = subtenon 20-mg triamcinolone injection group.

## Discussion

Our results suggested that a single subtenon 20-mg triamcinolone injection had comparable therapeutic efficacy to 0.1% dexamethasone eye drops in controlling ocular inflammation after uneventful phacoemulsification. All patients had maximal inflammation on POD1 and gradually decreased over time. However, the aqueous flare remained slightly higher than baseline in both groups at the end of the study.

Although there was no significant difference in postoperative inflammation control between two groups, the patients who received subtenon steroid injection tended to have less inflammation on POD1 than those with steroid eye drops, as seen in the mean flare from LFCM. This finding reflected the difference in starting time between administration routes. The patients in the Dexa group started corticosteroids eye drops one day following the cataract surgery, whereas those patients in the TA group received corticosteroids injection immediately after operation. However, the efficacy of the corticosteroids between the groups was comparable during the subsequent follow-up visits. These findings suggested that the effect of a single subtenon triamcinolone injection lasted long enough to control inflammation postoperatively similar to the conventional treatment.

During the follow-up, we found that the mean cells in the Dexa group were significantly lower than in the TA group on POD90, however, this level is within the range of normal people according to the study by EI-Maghraby et al. They reported the mean cells from LFCM of 18.67 /mm^3^ in preoperative cataract patients using the same machine, FC-1000 laser flare-cell meter^17^. Therefore, the difference might be from the variation among individuals.

Our study showed similar results as the studies by Paganelli et al. in 2004 and 2009, and Khan et al. in 2016^13,15^ Paganelli et al. compared efficacy and safety between single subtenon 40- and 25-mg triamcinolone injection with 1% prednisolone acetate eye drops to control postoperative inflammation after phacoemulsification, while Khan et al. compared subtenon 40-mg triamcinolone injection with 0.1% dexamethasone eye drops. The peak inflammation was recorded in POD1 and comparably decreased over time in both groups. No substantial differences in efficacy between the two treatments were reported, and no serious adverse events were observed in each group. Nevertheless, they assessed postoperative inflammation subjectively using slit-lamp biomicroscopy, but we confirmed the degree of inflammation using objective evaluation with LFCM.

In 2006, Negi et al.^14^ quantitatively compared the efficacy of a single subtenon injection of triamcinolone with 0.1% betamethasone sodium phosphate eye drops following cataract surgery and found no overall difference between the two groups. Nonetheless, their protocol had started with the same dose as ours (20-mg triamcinolone) in the injection group before being changed to a 30-mg triamcinolone injection because of inadequate treatment effect. They found excessive postoperative inflammation in 4 out of the first ten patients randomized to the 20-mg injection. Unlike their findings, none of our patients experienced severe inflammation despite receiving the 20-mg triamcinolone. The reason might be the improvement of phacoemulsification technology which provides a better postoperative outcome. Furthermore, the different IOL materials have different biocompatibility. While Negi et al. used silicone IOL in their study, we used hydrophobic acrylic IOL, resulting in better control of postoperative inflammation under the low dose triamcinolone.

An IOP elevation occurs commonly after corticosteroids therapy. There were reports of high IOP spikes in the previous studies 25- to 40-mg subtenon injections^14,20,21^. We tried to use a smaller dose of 20-mg triamcinolone to control postoperative inflammation in this trial. Subtenon triamcinolone injection usually had a maximum effect at three to six weeks and lasted no longer than eight weeks. Our study had a follow-up period of 90 days which would be long enough to detect such complications. However, we found no difference in overall numbers and levels of increased IOP between injection and eye drops groups. Only one patient in our study (eye drops group) had IOP higher than 30 mmHg. All patients with high IOP were successfully treated with 0.5% timolol eye drops for one month.

Our results are comparable to those of Paganelli et al.^15^ They reported no patient having IOP more than 25 mmHg after 40 mg triamcinolone injection for up to 2 months of follow-up visits. Moreover, in the triamcinolone group, a considerably lower intraocular pressure was noted at PODs 3, 7, 14, and 28 compared to the eye drops group. However, a study by Helm and colleagues ^22^ reported IOP rising over 21 mmHg in six patients (30%) at three weeks after the 40 mg triamcinolone injection with four patients underwent filtration surgery. Lafranco and colleagues^23^ also reported 6 of 64 eyes with uncontrolled IOP requiring filtering surgery after subtenon 40 mg triamcinolone injection. Byun et al.^12^ observed the incidence of high IOP in 18 of 159 eyes after subtenon 40-mg triamcinolone injection, but the IOP was well controlled with the IOP lowering eye drops. Negi et al.^14^ also showed the higher IOP after 30 mg triamcinolone injection with four eyes had IOP above 30 mmHg on POD1; however, the difference was not substantial.

Ocular tolerance in terms of pain and discomfort was not different between the two groups, although patients in injection groups reported less pain and discomfort on POD1. The trend went the same way for conjunctival injection. The responses reflected the postoperative treatments. Unlike the immediate postoperative subtenon injection, the participants started eye drops one day after surgery. As a result, the anti-inflammatory effect began later in the Dexa group.

There were no differences in visual outcomes and other adverse events between groups. No cystoid macular edema or endophthalmitis occurred in our study. A small number of patients developed pigment dispersion after phacoemulsification, which precluded the laser cell reading. There were no complications from subtenon triamcinolone injection detected like those reported previously, including inadvertent injection into choroidal or retinal circulation, globe penetration, retinal vascular occlusion, blepharoptosis, proptosis, orbital fat prolapses, orbital fat atrophy, delayed hypersensitivity reactions, and strabismus^24–29^. Though this study might not have enough participants to detect rare complications.

Our study had some limitations. First, we did not have a placebo for triamcinolone injection in the Dexa group. However, during the postoperative period, patients in both groups had received subconjunctival gentamicin injections, which could mask which group they were assigned to. Second, the small sample size made the results inconclusive. We could not find the differences in terms of efficacy and safety between the two comparison groups. A non-inferiority or an equivalent large multicenter trial with a larger sample size should be conducted in the future to confirm the findings. From post hoc analysis, the sample size to detect the difference in the proportion of zero ACI score between groups on POD 28 was 1514 in each group with a power of 80% and a type one error rate of 0.05. Moreover, expanding the usage of subtenon triamcinolone to complicated cases might better exhibit their benefit.

In conclusion, a single subtenon injection of 20-mg triamcinolone demonstrated comparable safety and efficacy to 0.1% dexamethasone eye drops in controlling postoperative inflammation. The results from our study should benefit the patients and the ophthalmologists to broaden the choices of treatment, especially for patients who have difficulty in putting eye drops or to follow-up. According to the literature, 20-mg of triamcinolone appears to be the lowest dose to control inflammation after phacoemulsification.

## Data Availability

The datasets used and analyzed during the current study available from the corresponding author on reasonable request.
